# Proof-of-Concept Study on the Use of Tangerine-Derived Nanovesicles as siRNA Delivery Vehicles toward Colorectal Cancer Cell Line SW480

**DOI:** 10.3390/ijms25010546

**Published:** 2023-12-30

**Authors:** Nima Rabienezhad Ganji, Ornella Urzì, Vincenza Tinnirello, Elisa Costanzo, Giulia Polito, Antonio Palumbo Piccionello, Mauro Manno, Samuele Raccosta, Alessia Gallo, Margot Lo Pinto, Matteo Calligaris, Simone Dario Scilabra, Maria Antonietta Di Bella, Alice Conigliaro, Simona Fontana, Stefania Raimondo, Riccardo Alessandro

**Affiliations:** 1 Dipartimento di Biomedicina, Neuroscienze e Diagnostica Avanzata, Università degli Studi di Palermo, 90133 Palermo, Italy; nima.rabienezhadganji@unipa.it (N.R.G.); ornella.urzi@unipa.it (O.U.); vincenza.tinnirello@unipa.it (V.T.); elisa.costanzo01@unipa.it (E.C.); m.antonietta.dibella@unipa.it (M.A.D.B.); alice.conigliaro@unipa.it (A.C.); simona.fontana@unipa.it (S.F.); riccardo.alessandro@unipa.it (R.A.); 2 Dipartimento di Scienze e Tecnologie Biologiche Chimiche e Farmaceutiche, Università degli Studi di Palermo, 90128 Palermo, Italy; giulia.polito@unipa.it (G.P.); antonio.palumbopiccionello@unipa.it (A.P.P.); 3 Institute of Biophysics, National Research Council of Italy, 90146 Palermo, Italy; mauro.manno@cnr.it (M.M.); samuele.raccosta@ibf.cnr.it (S.R.); 4 Research Department, IRCCS-ISMETT (Istituto Mediterraneo per i Trapianti e Terapie ad Alta Specializzazione), 90127 Palermo, Italy; agallo@ismett.edu; 5 Proteomics Group of Fondazione Ri.MED, Department of Research IRCCS-ISMETT, via Ernesto Tricomi 5, 90145 Palermo, Italymcalligaris@fondazionerimed.com (M.C.);

**Keywords:** plant-derived nanovesicles, drug delivery, electroporation, siRNA

## Abstract

In the last years, the field of nanomedicine and drug delivery has grown exponentially, providing new platforms to carry therapeutic agents into the target sites. Extracellular vesicles (EVs) are ready-to-use, biocompatible, and non-toxic nanoparticles that are revolutionizing the field of drug delivery. EVs are involved in cell–cell communication and mediate many physiological and pathological processes by transferring their bioactive cargo to target cells. Recently, nanovesicles from plants (PDNVs) are raising the interest of the scientific community due to their high yield and biocompatibility. This study aims to evaluate whether PDNVs may be used as drug delivery systems. We isolated and characterized nanovesicles from tangerine juice (TNVs) that were comparable to mammalian EVs in size and morphology. TNVs carry the traditional EV marker HSP70 and, as demonstrated by metabolomic analysis, contain flavonoids, organic acids, and limonoids. TNVs were loaded with DDHD1-siRNA through electroporation, obtaining a loading efficiency of 13%. We found that the DDHD1-siRNA complex TNVs were able to deliver DDHD1-siRNA to human colorectal cancer cells, inhibiting the target expression by about 60%. This study represents a proof of concept for the use of PDNVs as vehicles of RNA interference (RNAi) toward mammalian cells.

## 1. Introduction

Drug delivery refers to the vehiculation of a substance to a specific area, tissue, or cell where its subsequent controlled release will ensure the greatest efficiency [1]. In the last years, innovative approaches and procedures based on drug delivery have emerged for the treatment of several diseases, including cancer. It is well known that most cancer treatments are associated with a wide range of side effects and can be more challenging and complicated for older adults, such as a poor quality of life, long-term hospitalization, and death [2]. The increased effectiveness of the treatment at the desired site and the lack of off-target effects and drug toxicity are the main benefits of a drug delivery system [3]. However, some compounds, such as therapeutic RNA, do not enter cells easily due to anionic charge and their susceptibility to RNases, thus making the use of vehicles essential [4]. One of the frequently used systems for drug delivery is represented by liposomes employed, for example, for the delivery of doxorubicin to cancer cells [5]. Other drug delivery systems include micelles, dendrimers, nanocapsules, and peptide-based nanoparticles [1]. Although some of these vehicles have been successfully translated into clinical applications [6], their use is still hampered due to cytotoxicity and immunogenicity.

Extracellular vesicles (EVs) have several advantages over conventional synthetic carriers, such as low toxicity, high biocompatibility, and low immunogenicity [7,8]. EVs are lipid bilayer-delimited particles that are naturally released from cells and take part in cell–cell communication [9,10]. EVs can transfer protein, nucleic acids, and other biomolecules into target cells, thus acting as genuine delivery carriers [11,12,13,14]. The ability of EVs to act as nanocarriers for drug delivery has been proven, and they have been effectively used to transport chemotherapeutic drugs [15,16]. However, drug and nucleic acid loading into EVs is still a challenging procedure.

Currently, several techniques have been applied to load various drugs into EVs, such as sonication, electroporation, transfection, incubation, extrusion, saponin-assisted loading, transgenesis, freeze–thaw cycles, thermal shock, the pH gradient method, and hypotonic dialysis [17]. Specifically, for the loading of RNA molecules (siRNA, mRNAs, and miRNAs), Han et al. demonstrated that electroporation has an increased efficiency [18]. In line with this finding, other studies supported the idea that the electroporation approach is one of the best methods to load RNA inside EVs [19,20].

In EV-based drug delivery systems, the amount of siRNA incorporated into the EVs is crucial. Evaluation of the loading rate of siRNAs into EVs using electroporation revealed that this method can promote siRNA aggregation, which may lead to an overestimation of the loading efficiency; to minimize the observed siRNA aggregation, EDTA was added [21].

Most of the data are focused on animal-derived EVs; however, in recent years, numerous studies have been carried out on plant nanovesicles (PDNVs). PDNVs can be purified from different parts of the plant, such as roots, leaves, fruits, and seeds [22,23], and also participate in intercellular interactions across different kingdoms [24,25]. We demonstrated that nanovesicles isolated from lemon juice have anti-cancer effects by activating apoptosis [26]. Another study conducted in 2018 showed that EVs extracted from ginger can be used to transfer therapeutic siRNAs to tumor cells and inhibit tumor growth [27]. However, very few studies about strategies for loading siRNAs into plant nanovesicles are currently available.

In the present study, we isolated and characterized nanovesicles from tangerine juice (TNVs) and developed a protocol to load into TNVs exogenous siRNAs targeting DDHD1, whose overexpression in colon cancer cells is associated with an increase in the proliferation rate [28]. 

## 2. Results

### 2.1. Isolation and Characterization of Tangerine-Derived Nanovesicles (TNVs)

**Figure 1:**
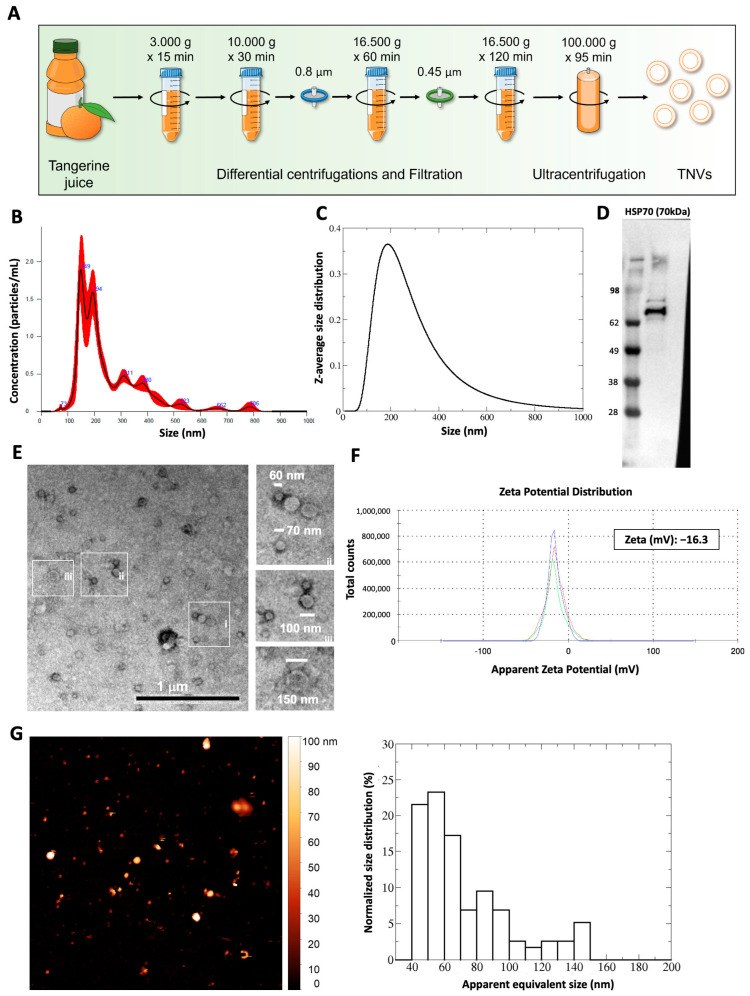
(**A**) Schematic illustration of the protocol of TNV isolation. (**B**) Size distribution of TNVs obtained through NTA. (**C**) Size distribution of TNVs obtained through DLS. (**D**) Western blot analysis of HSP70 in TNVs. (**E**) Representative image and relative magnifications of transmission electron microscopic analysis showing the heterogeneity in size of the isolated TNVs. (**F**) Zeta potential of TNVs; the Zeta value (mV) is the mean of three records. (**G**) Representative AFM image (5 × 5 µm^2^) of TNVs (left panel) and the related statistical analysis (right panel).

TNVs were isolated from the fruit juice using the differential centrifugation method as shown in Figure 1A. Once isolated, TNVs were characterized by using nanoparticle tracking analysis (NTA), Western blotting, a transmission electron microscope (TEM), and an atomic force microscope (AFM). The NTA analysis showed that the TNVs had a heterogeneous size distribution, with a mean of 255 nm ± 18 nm and a mode of 180 nm ± 14 nm (Figure 1B). A DLS analysis confirmed these results, showing a size distribution with a z-average size of 236 ± 3 nm, a mode of 187 ± 3 nm, and a polydispersity index of 0.26 (Figure 1C). To gain more insight into the composition of plant nanovesicles and to search for relevant markers, we performed a proteomic analysis on the TNVs. Among the identified proteins (with the minimal peptide length set at 6 and the false discovery rate set at 0.01%), we found the heat shock proteins HSP70 and HSP90, tetraspanins, annexins, ABC transporters, and glyceraldehyde-3-phosphate dehydrogenase (Supplementary Table S1). These proteins belong to different families identified in mammalian vesicles [10] and in plants, as also reviewed in a letter to the editor by Pinedo and colleagues in 2021 [29]. Through Western blotting, the presence of HSP70, a protein described as a marker of both animal and plant EVs [30], was confirmed (Figure 1D). The transmission electron microscope analysis showed that after negative staining, the vesicles presented the morphologically typical cup or nearly round shapes (Figure 1E). The sizes were observed within a range between 40 and 150 nm, which was consistent with the characteristics of small vesicles [10]. In line with nanovesicles from other plant species, the TNVs had a negative Z-potential of −5.94 mV (Figure 1F). The AFM analysis showed that the apparent width of the TNV population ranged between 40 and 110 nm (Figure 1G). Since AFM sampling depends upon the particle number concentration, the size distribution measured via AFM indicated lower values with respect to the one measured via DLS and NTA, which were affected by the contributions of larger particles.

**Figure 2:**
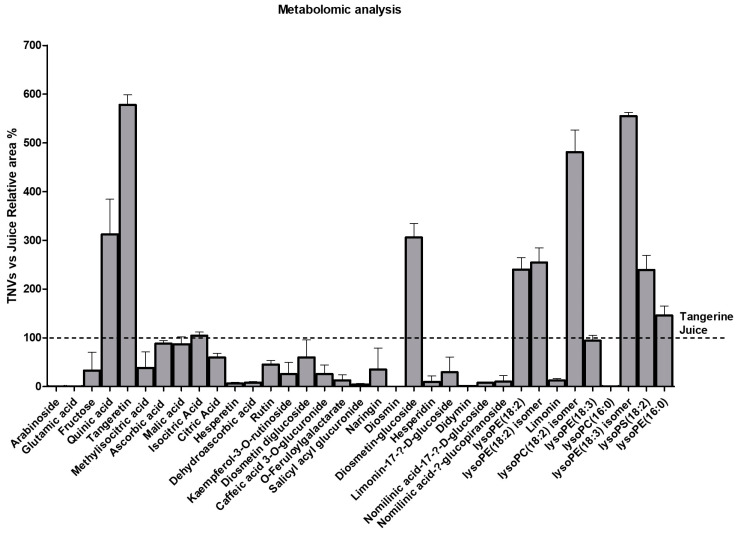
Metabolomic characterization of TNVs. Relative area % of metabolites into TNVs versus juice (*n* = 2).

Furthermore, we performed a metabolomics analysis of the TNVs and identified 35 compounds, which are summarized in Table 1. The detected compounds could be classified into different families such as organic acids, flavonoids, limonoids, cinnamic acid derivatives, lysophospholipids, carbohydrates, and a salicylic acid derivative. All compounds were also present in the tangerine juice, but interestingly, some of them were concentrated into nanovesicles (Figure 2). Tengeretin, quinic acid, diosmetin diglucoside, and lysophospholipids (except lysoPC(16:0)) were substantially concentrated in the TNVs. Organic acids were unchanged, while limonoids and other flavonoids presented lower concentrations in the TNVs compared to juice. 

To test the safety of non-modified TNVs on normal and cancer cell lines, we selected HS5 (human stromal cells), HDFα (human dermal fibroblasts), THLE2 (human hepatocyte), SW480 (human colorectal cancer cells), and Caco-2 (human colorectal adenocarcinoma cells) cells and treated them with increasing doses of TNVs (1, 5, 10, and 25 μg/mL). Following 24 and 48 h of treatment with TNVs, we analyzed the cell viability of the target cells by using an MTT assay, and we observed that the TNVs did not alter the cell viability (Supplementary Figure S1A). To ensure that the colorectal cancer cells were able to internalize TNVs, we labeled them with PKH-26 and treated SW480 for 2 h. Also, we monitored the TEV internalization in the Caco-2 cells that, although originally derived from colon carcinoma, has been widely used as a model of the intestinal epithelial barrier for their differentiation ability and low aggressiveness [16,31]. A fluorescence microscopy analysis revealed that both cell lines internalized the vesicles; however, the signal was higher in SW480, the most aggressive cell line (Supplementary Figure S1B); this is a key element in the development of targeted therapies that aim to maximize treatment efficacy and minimize side effects.

### 2.2. TNVs Can Be Loaded with siRNA through Electroporation

**Figure 3:**
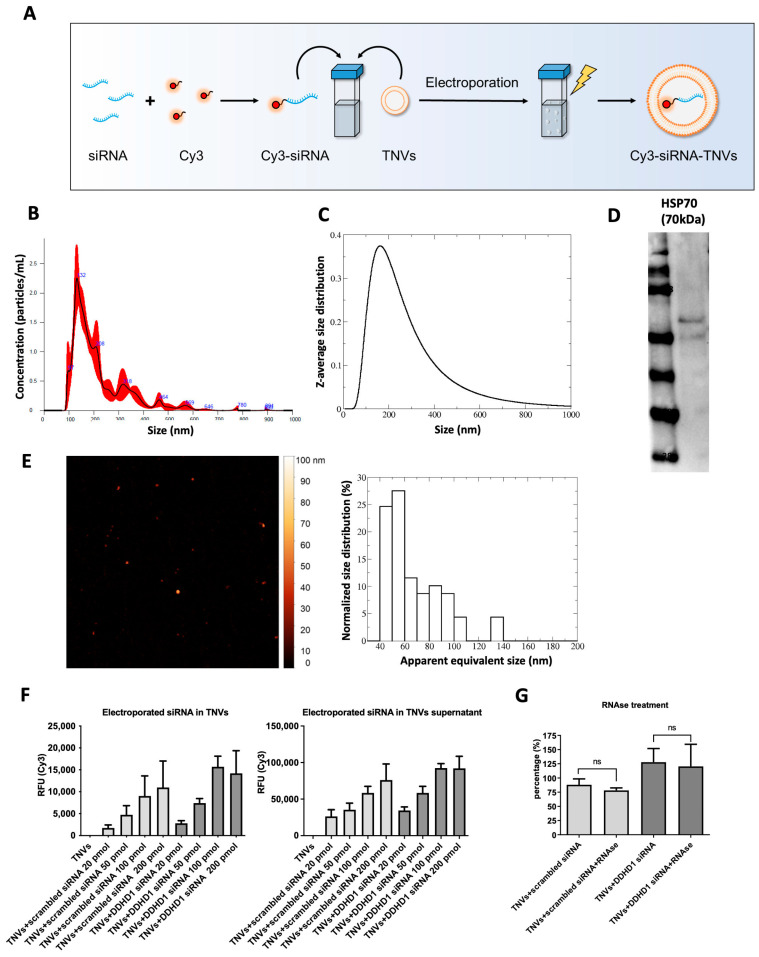
Cy3-siRNAs loading in TNVs through electroporation. (**A**) Schematic illustration of the protocol of TNV electroporation. (**B**) Size distribution of electroporated TNVs obtained through NTA. (**C**) Size distribution of electroporated TNVs obtained through DLS. (**D**) Western blot analysis of HSP70 in electroporated TNVs. (**E**) Representative AFM image (5 × 5 µm^2^) of electroporated TNVs (left panel) and the related statistical analysis (right panel). (**F**) The measurement of the fluorescent signal of the Cy3-siRNA TNV pellet and supernatant was carried out with the plate reader (*n* = 2–3). (**G**) The measurement of the fluorescent signal of the Cy3-siRNA TNVs treated with RNase was carried out with the plate reader (*n* = 3).

To test the possibility of using TNVs as natural nanovehicles for drug delivery, we selected the siRNA for DDHD1 since we previously reported that DDHD1 is involved in colorectal cancer cell proliferation and survival [28]. First, we labeled scrambled and DDHD1-siRNAs with Cy3 to follow the tracking of siRNA in the following experiments. Once we labeled the DDHD1-siRNA, we electroporated 25 μg of TNVs with different amounts (20, 50, 100, or 200 pmol) of Cy3-scrambled-siRNA or Cy3-DDHD1-siRNA (Figure 3A). To confirm that the electroporation protocol did not alter the TNVs’ structure and size, we performed NTA, DLS, Western blotting, and AFM. As shown in Figure 3B, the size distribution measured via NTA was similar to the result shown in Figure 1B. The electroporated TNVs had a mean size of 216 nm ± 9 nm and a mode of 143 nm ± 7 nm. Analogous results were obtained via DLS (Figure 3C), which displayed a z-average size of 214 ± 2 nm, a mode of 163 ± 2 nm, and a polydispersity index of 0.31. While the particle concentration measured via NTA was unchanged between the unloaded and electroporated TNVs, the total scattered intensity measured via DLS, which was proportional to the concentration, was lowered by one order of magnitude upon electroporation. Since the DLS was still able to capture the entire range in the size distribution, this observation warned us that the electroporation process may have induced the rupture/aggregation of some TNVs that were then removed from the solution via centrifugation; indeed, the samples were mildly centrifuged before the DLS experiments, which is the standard procedure for dust removal. Through Western blotting, the presence of HSP70 was detected in the electroporated TNVs (Figure 3D). The AFM images (Figure 3E) showed similar but less numerous objects to those in Figure 1G (2.3 TNVs/µm^2^ for TNVs vs. 1.4 TNVs/µm^2^ for electroporated TNVs), in accordance with the DLS measurements. Overall, these data demonstrated that the selected electroporation protocol did not impair the structure of TNVs.

To investigate the yield of the Cy3-siRNAs loaded in TNVs, we measured the fluorescent signal of both the Cy3-siRNA TNVs and of the supernatant obtained after the centrifugation to separate the TNVs from the electroporation buffer (see Section 4). As shown in Figure 3F, the fluorescent signal was proportional to the amount (pmol) of Cy3-siRNA loaded in the TNVs. Furthermore, we also detected fluorescence in the supernatant, suggesting that the loading efficiency was not 100%. The ratio between the amount of Cy3-siRNA in the supernatant and that in TNVs showed that our loading efficiency was 13%. 

Finally, to confirm that the Cy3-siRNA was internalized in the TNVs and not just attached to their surface, we removed non-internalized RNAs via RNase treatment and then measured the fluorescent signal. As shown in Figure 3G, the fluorescent signal of the RNase-treated TNVs was comparable to the signal of the untreated TNVs, demonstrating that our electroporation protocol determined the internalization of siRNA inside the TNVs; the slight decrease in the fluorescence signal in the RNase-treated TNVs may suggest that a small amount of siRNA was on the TNVs’ surface. Overall, these data demonstrated that we successfully loaded DDHD1-siRNA into TNVs through electroporation.

### 2.3. TNVs Delivered DDHD1-siRNA in Target Cells and Affected DDHD1 Expression

**Figure 4:**
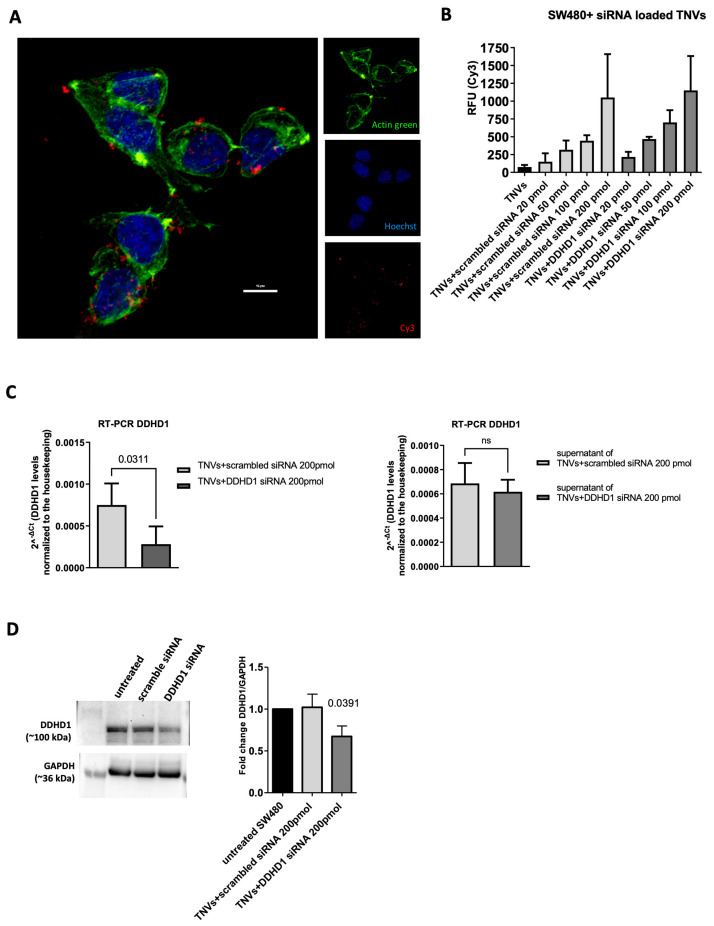
TNVs transfer DDHD1-siRNA into target cells, thus downregulating DDHD1 expression. (**A**) Confocal microscopy analysis of SW480 cells incubated for 24 h with Cy3-DDHD1-siRNA TNVs (blue = nuclei, green = actin, and red = Cy3-siRNA). (**B**) Cy3 fluorescence quantification of SW480 cells incubated for 24 h with TNVs, scrambled-siRNA TNVs, and DDHD1-siRNA TNVs (*n* = 3). (**C**) The gene expression level of DDHD1 in SW480 cells treated with scrambled-siRNA TNVs and DDHD1-siRNA TNVs pellets (left panel, *n* = 4) and supernatants (right panel, *n* = 5) for 48 h. Values are plotted as the mean ± SD of the level of the target gene normalized to the housekeeping; “ns” is not significant. (**D**) Western blot analysis of DDHD1 protein levels in SW480 cells treated with TNVs, scrambled-siRNA TNVs, and DDHD1-siRNA TNVs (*n* = 3).

To verify whether the Cy3-siRNA TNV complex was internalized by human tumor cells, we incubated SW480 cells with Cy3-DDHD1-siRNA TNVs and analyzed them with a confocal microscope. As shown in Figure 4A, the Cy3-DDHD1-siRNA TNVs were internalized by the target cells after 24 h of incubation. To confirm the confocal microscope analysis, we treated the SW480 cells with Cy3-scrambled-siRNA TNVs and Cy3-DDHD1-siRNA TNVs for 24 h; at the end of the treatment, the cell culture medium was removed, cells were washed twice in PBS to remove the non-internalized vesicle–siRNA complex, fresh PBS was added, and then the fluorescent signal was measured at glomax. We observed that the fluorescent signal was directly proportional to the amount (pmol) of siRNA loaded into the TNVs (Figure 4B).

Once we assessed that the TNVs were able to successfully transfer siRNAs into the target cells, we tested the function of siRNA-TNV complex on the modulation of DDHD1 in colorectal cancer cells. The SW480 cells were treated with both the DDHD1-siRNA TNV pellet and the supernatant for 48 h, and then we analyzed the gene expression of DDHD1. The obtained results showed that the treatment with the DDHD1-siRNA TNV supernatant did not affect the DDHD1 gene expression. On the other hand, the treatment with the DDHD1-siRNA TNV pellet downregulated DDHD1 gene expression in the SW480 cells (Figure 4C); this reduction was comparable to that observed in SW480 transfected with DDHD1-siRNA using the commercially available transfection reagent HiPerFect (Supplementary Figure S1C). The downregulation of DDHD1 was confirmed also at the protein level: the Western blot analysis demonstrated that the protein level of DDHD1 in the SW480 cells treated with a DDHD1-siRNA TNV pellet was lower compared to the control condition and cells treated with a DDHD1-siRNA TNV supernatant (Figure 4D). The TNV-siRNA-mediated downregulation of DDHD1 revealed a trend of reduction in the cell viability of 17% and 23% in cells treated with TNVs respectively loaded with 200 and 400 pmoli of DDHD1 siRNA (Supplementary Figure S1D). 

## 3. Discussion

Studies on the impacts and use of plant nanovesicles are quickly increasing. The potential to use plant nanovesicles as drug delivery systems for the treatment of various diseases, such as cancer and inflammation, has also been studied in recent years. [32,33,34,35,36]. The ability of plant nanovesicles to be internalized into mammalian cells is the primary factor contributing to the feasibility of using them as therapeutic carriers [37,38]. Moreover, their lack of potential toxicity for humans [39], their biocompatibility, and their biodegradability make them attractive candidates for drug delivery.

According to the findings of our study, homogeneous populations of nanovesicles with an EV-like size, shape, and protein content were isolated from tangerine juice. We have also observed that TNVs are abundant in lipids, limonoids, and flavonoid derivatives. Many of these compounds have been extensively investigated for their possible therapeutic role in several cancer types, including breast, lung, prostate, and colon cancer. Among them, tangeretin [40,41,42], hesperetin [43,44], rutin [45,46], and naringenin [46], which are already known for their considerable anti-cancer properties, give TNVs their intrinsic valuable qualities, which are intriguing for future studies. 

In line with previous findings showing that nanovesicles derived from plants do not affect normal cell growth [47,48], here we found that TNVs had no cytotoxic effects on different normal cell lines; in our study, TNVs at the tested concentration did not even alter the viability of colon cancer cells. These results highlighted the possibility of using them as drug vehicles. Notably, our observations revealed a higher internalization of TNVs by the aggressive colorectal cancer cell lines SW480 when compared to Caco-2 cells. In the context of utilizing TNVs as drug delivery vehicles, this process may enhance the targeted delivery of therapeutic agents to cancer cells, minimizing the impact on healthy tissues and resulting in a more effective and selective therapeutic outcome. 

Given their biocompatibility and safety, several studies have been conducted to identify a successful method for drug loading in plant nanoparticles [33,37,49,50]. Nowadays, sonication and co-incubation are the most used approaches for drug loading in plant nanoparticles [32,39,51,52]. For instance, Garaeva et al. loaded grapefruit-derived nanovesicles with bovine serum albumin and heat shock protein 70 through a sonication technique [38]. Other groups used co-incubation methods to load nanoparticles isolated from aloe [49], acerola [50], and lemon [37]. To our knowledge, this is the first study in which electroporation was used for siRNA loading in plant nanoparticles. Indeed, before selecting electroporation as an eligibility method for TNV loading, we also tried co-incubation and sonication; however, neither of these two approaches allowed us to effectively load the nanovesicles. This encouraged us to draw attention to the distinctive characteristics of nanoparticles from various plant matrices, such as diverse lipid compositions [53], thus explaining the different loading approaches that have been used.

In this study, we effectively loaded siRNA into TNVs using the electroporation method; the transfection efficiency was found to be 13%. In this regard, prior research showed that our findings were consistent with the transfection effectiveness of the electroporation loading technique for animal nanovesicles [54,55,56]. In 2022, Zhang et al. investigated the effect of miR-665-loaded EVs on osteosarcoma. By using electroporation, they loaded EVs with miR-665; this complex can prevent the progression of osteosarcoma in vitro and in vivo [57]. Another study aimed to repair spinal cord injury (SCI) by loading siRNA in engineered nanovesicles through electroporation. Overall, EVs-siRNA targeted the injured spinal cord in SCI mice and elicited significant functional recovery [58]. To study the anti-cancer effect of FU-5 in colon cancer, FU-5 and miR-21 have been electroporated into EVs. In the last study, the researchers found that the EV-based FU-5 and miR-21 systems can effectively facilitate cellular uptake and that the complex reduces tumor proliferation and increases apoptosis [59].

Here, we demonstrated that the TNV-siRNA complex has functional properties. We demonstrated that DDHD1-siRNA was successfully transferred into target cells by TNVs, thereby reducing DDHD1 expression.

## 4. Materials and Methods

### 4.1. Tangerine Nanovesicles Isolation

TNVs were isolated by using differential centrifugation, filtration, and ultra-centrifugation of tangerine juice (*Citrus reticulata* Blanco) obtained from the company Agrumaria Corleone s.p.a. (Palermo, Italy). Given the citrus fruit’s seasonality, the annually produced fresh juice was frozen at −20 °C to have the possibility to work over the year; indeed, the experiments reported in this study were carried out using the juice produced in the same year. After thawing at 4 °C, the juice was centrifuged at 3000× *g* for 15 min and 10,000× *g* for 30 min. The supernatant was filtered at 0.8 μm and centrifuged at 16,500× *g* for 1 h, then the obtained supernatant was filtered with a 0.45 μm pore filter and centrifuged at 16,500× *g* for 2 h. The resulting supernatant was then ultra-centrifuged at 100,000× *g* for 1 h and 45 min in a Type 70 Ti fixed-angle rotor. The pellet containing the vesicles was washed and suspended in phosphate-buffered saline (PBS). TNV protein quantification was determined with the Bradford assay (Pierce, Rockford, IL, USA). On average, we recovered 120–180 micrograms of vesicles from 240 mL of tangerine juice. 

### 4.2. Nanoparticle Tracking Analysis

The concentrations and size distribution of the tangerine vesicles were measured by using a nanoparticle tracking analysis (NTA) (NanoSight NS300, Malvern Instruments Ltd., Malvern, UK). The TNV samples were diluted 1:100 in PBS to reach the optimal concentration for instrument linearity. The particle size measurement was calculated on a particle-by-particle basis in 3 videos of 60 s to provide accuracy and statistics for each analysis under the following conditions: cell temperature—23.3–23.6 °C; syringe speed—30 µL/s. Recorded data were analyzed for the mean, mode, median, and estimated concentration of particles by using the in-build NanoSight Software NTA 3.3 with a detection threshold of 5. The hardware used were an embedded laser (45 mW at 488 nm) and a camera (sCMOS).

### 4.3. Dynamic Light Scattering

Exosome size distribution was determined via dynamic light scattering (DLS) experiments. The collected nanovesicle samples were diluted in Milli-Q water (2 times for untreated vesicles and 20 times for electroporated ones) and centrifuged at 1000× *g* for 10 min at 4 °C. The supernatant was placed at 20 °C in a thermostated cell compartment of a Brookhaven Instruments BI200-SM goniometer equipped with a He-Ne laser (JDS Uniphase 1136P) tuned at 633 nm and a single-pixel photon-counting module (Hamamatsu C11202-050). The scattered light intensity and its autocorrelation function g_2_(t) were measured simultaneously at a scattering angle of 90° by using a Brookhaven BI-9000 correlator. The intensity autocorrelation function g_2_(t) is related to the size σ of diffusing particles and to their (z-averaged) size distribution P_q_(σ) by the relation g_2_(t) = 1 + |β ∫ P_q_(σ) exp[−D(σ)q^2^t]|^2^, where β is an instrumental parameter, q = 4π ñ λ^−1^ sin[ϑ/2] is the scattering vector (with ñ being the refractive index of the medium), and D(σ) is the diffusion coefficient of a particle of hydrodynamic diameter σ determined by the Stokes–Einstein relation D(σ) = k_B_T [3πησ]^−1^, with T being the temperature, η the medium viscosity, and k_B_ the Boltzmann constant [60]. The size distribution P_q_(σ) was calculated by assuming that the diffusion coefficient distribution was shaped as a Schultz distribution, which is a two-parameter asymmetric distribution determined by the average diffusion coefficient $\bar{D}$ and the polydispersity index PDI [61,62].

### 4.4. Cell Culture

The human colorectal adenocarcinoma cell line SW480 was obtained from ATCC (Manassas, VA, USA) and was cultured in RPMI 1640 medium (Euroclone, Oxford, UK) supplemented with 10% fetal bovine serum (FBS, Euroclone), 2 mM L-glutamine (Euroclone), 100 U/mL penicillin, and 100 μg/mL streptomycin (Euroclone).

Human dermal fibroblasts (HDFα) were obtained from ATCC (Manassas, VA, USA) and were cultured in Fibroblast Basal medium (ATCC, Manassas, VA, USA) supplemented with Fibroblast Growth Kit-Low serum (ATCC, Manassas, VA, USA), 100 U/mL penicillin, and 100 µg/mL streptomycin (Euroclone, Oxford, UK).

Human stromal cells (HS5) were grown in high-glucose DMEM medium supplemented with 10% fetal bovine serum, 100 U/mL penicillin, and 100 μg/mL streptomycin (Euroclone, Oxford, UK). The Caco-2 cell line (ATCC, Manassas, VA, USA) was cultured in Eagle’s Minimum Essential Medium supplemented with 20% FBS.

The SV40 large T antigen-immortalized normal human liver epithelial cell line THLE2 (ATCC, Manassas, VA) was cultured in LHC-8 Basal Medium (Invitrogen, Carlsbad, CA, USA) supplemented with 70 ng/mL phosphoethanolamine, 5 ng/mL epidermal growth factor (EGF), 10% fetal bovine serum, 100 U/mL penicillin, and 100 μg/mL streptomycin (Euroclone, Oxford, UK). Cells were maintained in pre-coated flasks with a collagen coating composed of a mixture of 0.03 mg/mL bovine collagen type I (Advanced Biomatrix, San Diego region, CA, USA) and 0.01 mg/mL bovine serum albumin (Sigma-Aldrich, St. Louis, MO, USA).

The SW480, HDFα, HS5, Caco-2, and THLE2 cells were grown in a 37 °C humidified incubator with 5% CO_2_ and were passaged every 2–3 days.

### 4.5. Western Blotting

The protein lysates from TNVs and SW480 cells treated for 48 h with TNVs (25 μg/mL) or TNVs (25 μg/mL) loaded with 200 pmol of scrambled siRNA or DDHD1-siRNA were analyzed by using SDS-PAGE followed by Western blotting. The antibodies used in the experiments were anti-HSP70 (Agrisera, Vännäs, Sweden), anti-DDHD1 (Novus Biologicals, Bio-Techne SRL Milan, Italy), and anti-GAPDH (Santa Cruz Biotechnology, Dallas, TX, USA). Membranes were incubated with HRP-conjugated secondary antibody (Thermo Fisher Scientific, Cambridge, MA, USA), and the chemiluminescent signal was detected with Chemidoc (Biorad, Milan, Italy).

### 4.6. Transmission Electron Microscopy

The isolated TNVs resuspended in PBS were identified via transmission electron microscopy studies using negative staining. A 10 μL aliquot of TNV suspension was dropped onto 200 mesh carbon-coated EM grids, followed by standing at ambient temperature for 10 min. After washing with ddH_2_O, the samples were fixed for 5 min in 1% glutaraldehyde. Subsequently, the TNVs were negatively stained with a 2% aqueous solution of phosphotungstic acid. After air drying, the grids were visualized in a JEM 1400 Plus electron microscope (Jeol, Tokyo, Japan) operating at 80 kV.

### 4.7. Zeta Potential

The zeta potential of the TNVs was measured using a Zetasizer nano ZSP (Malvern). The TNVs (6 µg) were diluted in a solution containing 1 mM NaCl to a total volume of 0.6 mL; the pH of the solution was 7.8. The sample was transferred to the specific cuvette for zeta potential measurement (disposable capillary cell, DTS1070, Malvern).

### 4.8. Atomic Force Microscopy

Sample preparation: glass slides were functionalized according to the following treatment: (i) cleaned by immersion in boiling acetone for a few minutes, dried in a stream of high-purity nitrogen, and exposed to UV rays (30W Hg lamp) for 1 h to expose the hydroxyl groups of silica; (ii) treated with a 0.25 M (3-aminopropyl)-triethoxysilane (APTES) in chloroform solution for 5 min at room temperature and then rinsed with chloroform and dried with nitrogen; (iii) treated with 0.4 M glutaraldehyde aqueous solution for 5 min at room temperature and then rinsed with Milli-Q water and dried with nitrogen. A total of 30 µL of vesicle solution (diluted 200 times in PBS after centrifugation at 1000× *g* for 10 min at 4 °C) was deposited onto functionalized glass slides and incubated overnight. Vesicle imaging: samples were gently rinsed with PBS, and quantitative imaging AFM measurements were carried out in PBS by using a Nanowizard III scanning probe microscope (JPK Instruments AG, Berlin, Germany) equipped with a 15 µm z-range scanner and AC40 (Bruker, Billerica, MA, USA) silicon cantilevers (spring constant 0.1 N/m, typical tip radius 8 nm). The 5 × 5 µm^2^ images were acquired at a 512 × 512 pixel resolution and a force setpoint of 150 pN (z-length: 50 nm and 70 nm, pixel time: 5 ms and 7 ms for untreated and electroporated samples, respectively). In the electroporated sample, we set a higher z-length in response to the observed higher adhesion, which was likely caused by spurious sticky molecules or fragments due to electroporation. The cantilever was thermally calibrated by using the tool in JPK software version 4.2 [63]. The statistical analysis was performed using the grain analysis tools available in Gwyddion software ver. 2.62. In particular, the equivalent disc radius of the objects was computed, and the grains ascribed to nanovesicles were selected by using the following criteria: (a) height greater than 10 nm (limit case for two overlapped lipid bilayers) and (b) area greater than 15 pixels (to skip eventual spikes and objects that were too small to be recognized as nanovesicles).

### 4.9. Proteomics

#### 4.9.1. Sample Preparation

About 10 μg of TNV protein lysate was subjected to proteolytic digestion using a filter-assisted sample preparation (FASP) protocol with 10 kDa Vivacon spin filters (Sartorius, Göttingen, Germany).

Briefly, proteins were reduced via the addition of 1 M of dithiothreitol (DTT, Thermo Fisher Scientific) in 100 mM of Tris/HCl, 8 M of urea (pH 8.5) for 30 min at 37 °C. Proteins were then alkylated in 50 mM of iodoacetamide (IAA, Thermo Fisher Scientific) for 5 min at room temperature and washed twice in 100 mM of Tris/HCl and 8 M of urea (pH 8.0) at 14,000× *g* for 30 min. Proteins were digested with 0.2 μg of LysC (Promega, Madison, WI, USA) in 25 mM of Tris/HCl and 2 M of urea (pH 8.0) overnight and with 0,1 μg of trypsin (Promega) in 50 mM of ammonium bicarbonate for 4 h. The resulting peptides were desalted via stop-and-go extraction (STAGE) on reverse-phase C18 (Supelco Analytical Products, part of Sigma-Aldrich, Bellefonte, PA, USA) and eluted in 40 μL of 60% acetonitrile in 0.1% formic acid. Then, the volume was reduced in a SpeedVac (Thermo Fisher Scientific), and the peptides were resuspended in 20 μL of 0.1% formic acid. The peptide concentration was measured by using a NanoDrop microvolume spectrophotometer (Thermo Fisher Scientific), and 1 μg of peptides was applied to the LC-MS/MS analysis.

#### 4.9.2. LC-MS/MS

To achieve high sensitivity, a nanoLC system (Vanquish Neo UHPLC—part of Thermo Scientific) using an Acclaim PEPMap C18 column (25cm × 75 µm ID, Thermo Scientific, Waltham, MA, USA) was coupled online to an Exploris 480 mass spectrometer (Thermo Fisher Scientific). Peptides were separated using a 130 min binary gradient of water and acetonitrile containing 0.1% formic acid. Data-independent acquisition (DIA) was performed using a MS1 full scan (400 *m*/*z* to 1200 *m*/*z*) followed by 60 sequential DIA windows with an overlap of 1 *m*/*z* and the window placement optimization option enabled. Full scans were acquired with a 120,000 resolution, an automatic gain control (AGC) of 3 × 10^6^, and a maximum injection time of 50 ms. Afterwards, 60 isolation windows were scanned with a resolution of 30,000 and an AGC of 8 × 10^5^; the maximum injection time was set to auto to achieve the optimal cycle time. Collision-induced dissociation fragmentation was induced with 30% of the normalized HCD collision energy.

The data were analyzed with the software DIA-NN (version 1.8.1) by using a predicted library generated from an in silico digested Citrus sinensis (UP000027120) Uniprot reference database involving cuts at K* and R* with two missed cleavages allowed and a minimal peptide length set at 6. The false discovery rate for peptide and protein identification was set at 0.01%.

### 4.10. Reversed-Phase HPLC/MS

The water and acetonitrile were of HPLC/MS grade. The formic acid was of analytical quality. The analyses were performed by adapting a previously reported method [13]. The HPLC system was an Agilent 1260 Infinity. A reversed-phase Agilent Poroshell 120 EC-C18 column (50 mm × 3.0 mm; particle size 2.7 µm) with a Phenomenex C18 security guard column (4 mm × 3 mm) was used. The flow rate was 0.4 mL/min, and the column temperature was set to 30 °C. The eluents were formic acid–water (0.1:99.9, *v*/*v*) (phase A) and formic acid–acetonitrile (0.1:99.9, *v*/*v*) (phase B). The following gradient was employed: 0–10 min, linear gradient from 5% to 95% B; 10–12 min, reconditioning to 5% B; 12–15 min, 5% B isocratic. The injection volume was 15 µL. The eluate was monitored through MS TIC. Mass spectra were obtained on an Agilent 6540 UHD accurate-mass Q-TOF spectrometer equipped with a Dual AJS ESI source working in negative mode. N_2_ was employed as the desolvation gas at 320 °C and a flow rate of 7 L/min.

The nebulizer was set to 20 psig, the Sheat gas temperature was set at 295 °C, and the flow was 8 L/min. A potential of 2.6 kV was used on the capillary for negative ion mode. The fragmentor was set to 175 V. MS spectra were recorded in the 100–3200 *m*/*z* range. TNV samples for HPLC were analyzed “as is”. Reproducibility was verified with 3 replicates.

Mass spectral data were analyzed for metabolite annotation using MassHunter Qualitative Analysis B.06.00 and the Metlin database (METLIN—available online at https://metlin.scripps.edu/landing_page.php?pgcontent=mainPage (accessed on 15 November 2023).

### 4.11. Cell Viability Assays

Cell viability was assessed with the MTT assay (3-[4,5-dimethylthiazol-2-yl]-2,5 diphenyl tetrazolium bromide). The different cell lines (HDFα, HS5, THLE2, SW480, and Caco-2 cells) were seeded in triplicate in 96-well plates at a density of 10,000 cells/well and treated after 24 h with different doses of TNVs (1, 5, 10, and 25 μg/mL). The viability was evaluated at 24 and 48 h. Cells were also treated with 10% DMSO as a positive control of cell death. In addition, to determine the effects of siRNA-loaded TNVs on the viability of colorectal cancer cells, SW480 cell lines were seeded at 10,000 cells/well in duplicate in 96-well plates; 24 h after seeding, cells were treated with 25 μg/mL of TNVs previously loaded with 200 and 400 pmol of scrambled or DDHD1-siRNA. Cell viability was measured after 48 h. For all cell lines mentioned above, the absorbance was measured with an ELISA reader at 540 nm (Microplate Reader, BioTek, Winooski, VT, USA). Values are expressed as a percentage of cell growth versus control (untreated cells).

### 4.12. TNV Internalization in Human Cell Lines

TNVs were incubated with PKH26 Red Fluorescent Cell Linker Kits (Merck KGaA, Darmstadt, Germany) for 15 min, washed three times with PBS, and then resuspended in cell culture medium. Labelled TNVs were incubated for 2 h with the SW480 and Caco-2 cell lines at 37 °C. Following treatment, to verify the TNV internalization, cells were fixed with PFA 4% and permeabilized with TritonX-100. Cellular actin was stained with Actin Green (Molecular Probes, Life Technologies, Carlsbad, CA, USA), while nuclei were stained with Hoechst (Molecular Probes, Life Technologies, Carlsbad, CA, USA). The samples were analyzed by using confocal microscopy (Nikon A1, Amsterdam, The Netherlands). Data are expressed as the mean fluorescence intensity calculated on the cell surface.

### 4.13. siRNA Labeling

To evaluate the loading of siRNA inside the TNVs, labeling of scrambled and DDHD1-siRNA with Cy3 was performed using the Silencer siRNA labeling kit (Thermo Fisher Scientific). We prepared 5 pmol of Cy3-scrambled and Cy3-DDHD1-siRNA by following the manufacturer’s protocol. Briefly, Cy3 powder was dissolved with the reconstitution solution provided in the kit and incubated at 25 °C for 5 min. Then, a mixture containing siRNA, Cy3, labeling buffer, and H_2_O was prepared. The Cy3-siRNA mix was incubated while protected from light at 37 °C for 1 h, then 100% cold ethanol and 5 M of NaCl were added; the mix was vortexed, kept at −20 °C for 1h, and centrifuged for 20 min at 10,000× *g*. The supernatant was removed, while the pellet was washed in 70% ethanol; the mix was centrifuged for 10 min at 10,000× *g*. Finally, the supernatant was removed and the pellet was air-dried at room temperature for 15 min and then suspended in nuclease-free H_2_O. The fluorescence was measured with the GloMax^®^-Multi Detection System Protocol (Promega) to verify the labelling.

### 4.14. TNVs Electroporation

Electroporation was performed in a 0.2 cm gap sterile electroporation cuvette. TNVs (25 μg) were mixed with Cy3-scrambled-siRNA or Cy3-DDHD1-siRNA (20, 50, 100, or 200 pmol) and suspended in electroporation buffer (1.15 mM of potassium phosphate, 25 mM of potassium chloride, and 21% OptiPrep in PBS). The samples were electroporated at 2000 V for 0.8 ms and then incubated at 37 °C for 1 h to allow recovery of the nanovesicle membrane. Then, the sample was centrifuged at 14,000× *g* for 20 min to remove the non-loaded siRNA. The supernatant was collected, while the pellets containing electroporated TNVs were resuspended in PBS and used for cell treatment. Finally, the fluorescence of the complex Cy3-siRNA-TNVs and the supernatant were quantified with a GloMax^®^-Multi Detection System.

### 4.15. RNAse Treatment

Electroporated TNVs were treated with 10 μg/mL of Ribonuclease A (RNAse A) and incubated at 37 °C for 30 min. The sample was centrifuged at 14,000× *g* for 20 min; the supernatant was removed, while the pellets were washed and resuspended in PBS. The fluorescence of the TNVs-Cy3-siRNA complex was quantified with a GloMax^®^-Multi Detection System.

### 4.16. Confocal Microscopy

The SW480 cells were seeded at 100,000 cells/well (8 well chambers), and after 24 h, they were treated with Cy3-DDHD1-siRNA TNVs (25 µg of TNVs loaded with 200 pmol of scrambled siRNA) for 24 h. At the end of the experiment, the cells were fixed with PFA 4%, permeabilized with 0.1% TritonX-100, and stained with Actin Green (Molecular Probes, Life Technologies, Carlsbad, CA, USA) and Hoechst (Molecular Probes, Life Technologies, Carlsbad, CA, USA). The samples were analyzed by using confocal microscopy (Nikon A1, Amsterdam, The Netherlands).

### 4.17. Cell Transfection

Transfection of DDHD1-siRNA in SW480 was performed using the HiPerFect Transfection Reagent (Qiagen) according to the manufacturer’s instructions. Cells were plated in 24-well plates, and 24 h after seeding, they were transfected with 200 pmoles/mL of DDHD1-siRNA. Forty-eight hours after transfection, the cells were processed for RNA isolation and real-time PCR.

### 4.18. RNA Isolation and Real-Time PCR

SW480 cells were seeded in 12-well plates at 300,000 cells/well. After 24 h, the cells were treated with scrambled-siRNA TNVs and DDHD1-siRNA TNVs (25 μg of TNVs loaded with 200 pmol of scrambled or DDHD1-siRNA) and with respective supernatants collected after TNVs electroporation for 24 and 48 h. At the end of the treatments, the total RNA was extracted using an Illustra^TM^ RNA spin mini-RNA isolation kit according to the manufacturer’s instructions (GE Healthcare, Little Chalfont, Buckinghamshire, UK). The RNA was reverse-transcribed to cDNA using the High-Capacity cDNA Reverse Transcription kit (Applied Biosystems, Foster City, CA, USA). Then, RT-QPCR was performed in 48-well plates using a Step OneTM Real-time PCR System Thermal Cycling Block (Applied Biosystem). The sequences of the primers used for quantitative SYBR^®^Green real-time PCR were as Table 2:

Target transcript levels were normalized against the endogenous control GAPDH consistently expressed in all samples (ΔCt). For each condition, final values were expressed as the DDHD1 level normalized to the endogenous control (2^^−ΔCT^).

### 4.19. Statistical Analysis

Data are expressed as the mean ± SD of biological replicates. The statistical analysis was performed using GraphPad Prism software (Version 10.1.0 (316), GraphPad Software, Inc., La Jolla, CA, USA). The normal data distribution was assessed by using a Shapiro–Wilk test. When the data followed a normal distribution, the statistical significance of the differences was analyzed using a two-tailed Student’s *t*-test; otherwise, a non-parametric method (Mann-Whitney test) was used to compare the groups. A *p*-value ≤ 0.05 was considered significant.

## 5. Conclusions

To conclude, our research shows that plant-derived nanovesicles are suitable for use as drug delivery vehicles, suggesting they might become an innovative tool for the development of new therapeutics. Our study offers a ready-to-use platform for the delivery of therapeutic RNAi. Further studies are needed (i) to assess whether the electroporation protocol described here also can be applied to nanovesicles from different sources as well as to target different cell types and (ii) to elucidate whether an increase in siRNA doses loaded in TNVs correlates with changes in the cell phenotype.

## Figures and Tables

**Table 1:** Composition of TNVs. The table includes molecular formula, chemical class, retention time (Rt, min), and experimental and calculated *m*/*z* in negative ion mode.

	Compound	Molecular Formula	Chemical Class	ESI- [M-H]- (*m*/*z*) (*Teor*.)	ESI- [M-H]- (*m*/*z*) (*Exp*.)	Rt (min)
1	Arabinoside	C_10_H_15_N_5_O_5_	Carbohydrate	284.10	284.0970	3.33
2	Glutamic acid	C_5_H_9_NO_4_	Amino acid	146.0459	146.0459	3.42
3	Fructose	C_6_H_12_O_6_	Carbohydrate	179.0561	179.0575	3.45
4	Quinic acid	C_7_H_12_O_6_	Organic acid	191.0561	191.0551	3.77
5	Tangeretin	C_20_H_20_O_7_	Methoxylated flavonoid	371.1136	371.1153	3.89
6	Methylisocitric acid	C_7_H_10_O_7_	Organic acid	205.0354	205.0343	4.08
7	Ascorbic acid	C_6_H_8_O_6_	Organic acid	175.0248	175.0246	4.19
8	Malic acid	C_4_H_6_O_5_	Organic acid	133.0142	133.0143	4.29
9	Isocitric acid	C_6_H_8_O_7_	Organic acid	191.0197	191.0198	5.02
10	Citric acid	C_6_H_8_O_7_	Organic acid	191.0197	191.0192	5.26
11	Hesperetin	C_16_H_14_O_6_	Methoxylated flavanone	301.0714	301.0671	6.58
12	Dehydroascorbic acid	C_6_H_6_O_6_	Organic acid	173.0092	173.0086	7.10
13	Rutin	C_27_H_30_O_16_	Non-methoxylated flavonol glycoside	609.1461	609.1404	7.57
14	Kaempferol-3-O-rutinoside	C_27_H_30_O_15_	Non-methoxylated flavonol glycoside	593.1512	593.1461	7.67
15	Diosmetin diglucoside	C_28_H_32_O_16_	Methoxylated flavone glycoside	623.1618	623.1580	7.74
16	Caffeic acid 3-O-glucuronide	C_15_H_16_O_10_	Cinnamic acid derivative	355.0671	355.0645	7.95
17	O-feruloylgalactarate	C_16_H_18_O_11_	Cinnamic acid derivative	385.0776	385.0752	8.00
18	Salicyl acyl glucuronide	C_13_H_14_O_9_	Salicylate	313.0565	313.0548	8.08
19	Naringin	C_27_H_32_O_14_	Non-methoxylated flavanone glycoside	579.1719	579.1658	8.19
20	Diosmin	C_28_H_32_O_15_	Methoxylated flavonoid glycoside	607.1668	607.1636	8.21
21	Diosmetin glucoside	C_22_H_22_O_11_	Methoxylated flavone glycoside	461.1089	461.1079	8.25
22	Hesperidin	C_28_H_34_O_15_	Methoxylated flavonoid glycoside	609.1825	609.1781	8.27
23	Limonin-17-β-D-glucoside	C_32_H_42_O_14_	Limonoid	649.2502	649.2449	8.52
24	Didymin	C_28_H_34_O_14_	Methoxylated flavonoid glycoside	593.1876	593.185	8.65
25	Nomilinic acid-17-β-D-glucoside	C_34_H_48_O_16_	Limonoid	711.2870	711.2806	8.75
26	Nomilinic acid-β-glucopiranoside	C_34_H_46_O_15_	Limonoid	693.2764	693.2693	8.88
27	lysoPE(18:2)	C_23_H_44_NO_7_P	Lysophospholipid	476.2783	476.2738	9.50
28	lysoPE(18:2) isomer	C_23_H_44_NO_7_P	Lysophospholipid	476.2783	476.2752	10.24
29	Limonin	C_26_H_30_O_8_	Limonoid	515.1923	515.1863 (M+FA-H)	10.45
30	lysoPC(18:2) isomer	C_26_H_50_NO_7_P	Lysophospholipid	564.3307	564.3317 (M+FA-H)	10.70
31	lysoPE(18:3)	C_23_H_42_NO_7_P	Lysophospholipid	474.2626	474.2592	13.15
32	lysoPC(16:0)	C_24_H_50_NO_7_P	Lysophospholipid	540.3307	540.3284 (M+FA-H)	13.19
33	lysoPE(18:3) isomer	C_23_H_42_NO_7_P	Lysophospholipid	474.2626	474.2675	13.59
34	lysoPS(18:2)	C_24_H_44_NO_9_P	Lysophospholipid	520.2681	520.2585	13.85
35	lysoPE(16:0)	C_21_H_44_NO_7_P	Lysophospholipid	452.2783	452.2747	13.91

**Table 2:** Primer sequences used for RT-PCR.

Gene	Forward 5′ → 3′	Reverse 5′ → 3′
ACT	TCCCTTGCCATCCTAAAAAGCCACCC	CTGGGCCATTCTTCCTTAGAGAGAAG
DDHD1	TTTCTCAACCCAGCTAAAGAACCTA	TGATCCAACTCCAATGCAGAAT

## Data Availability

Data supporting the results present in this study are available upon reasonable request.

## Supplementary Materials

The following supporting information can be downloaded at: https://www.mdpi.com/article/10.3390/ijms25010546/s1.
