# Supplementary material for: Proof-of-Concept Study on the Use of Tangerine-Derived Nanovesicles as siRNA Delivery Vehicles toward Colorectal Cancer Cell Line SW480
Source: Int J Mol Sci. 2023 Dec 30;25(1):546. doi: 10.3390/ijms25010546 (PMC10779162; doi:10.3390/ijms25010546)
Supplement: Supplementary file 1 [file ijms-25-00546-s001.zip › Supplementary Figure S1.pdf]

# *Supplementary Material*

**Supplementary Figure S1:** (A) TNVs did not affect the viability of normal and cancer cells. MTT assay of HS5, HDFα, THLE2, SW480 and Caco-2 cells treated with different doses of TNVs (1, 5, 10, 25 μg/ml) for 24 and 48 h. SW480 and Caco-2 cells were also treated with 10% DMSO as positive control of cell death. (B) TNV internalization in SW480 and Caco-2 after 2 hours treatment; TNVs were stained with PKH26 (in red), actin with actin green (in green), and nuclei with Hoechst (in blue). Scale bars are 10μm. The histogram reports the mean fluorescence intensity (MFI) calculated based on cell surface. Data are represented as mean ±SD. (C) The gene expression level of DDHD1 in SW480 cells treated with DDHD1-siRNA TNV pellets or transfected with DDHD1-siRNA for 48 h using HiPerFect. Value are plotted as the mean ±SD of the level of the target gene normalized to the housekeeping. (n=4). (D) MTT assay of SW480 cells treated for 48 h with scrambled-siRNA TNVs and DDHD1-siRNA TNVs (n=2).

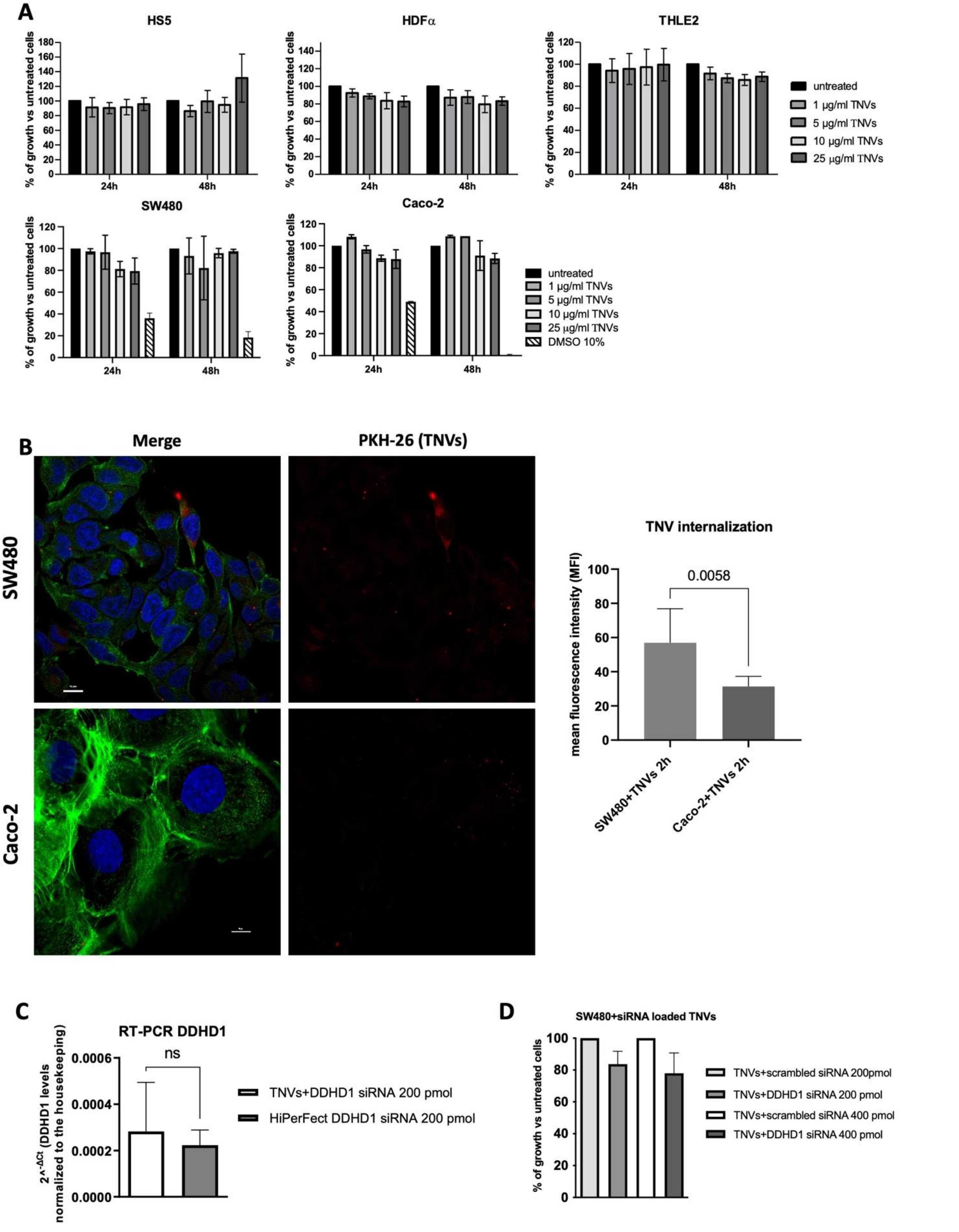
A
HS5
HDFα
THLE2
SW480
Caco-2
% of growth vs untreated cells
24h
48h
untreated
1 μg/ml TNVs
5 μg/ml TNVs
10 μg/ml TNVs
25 μg/ml TNVs
DMSO 10%
B
Merge
PKH-26 (TNVs)
SW480
Caco-2
TNV internalization
mean fluorescence intensity (MFI)
0.0058
SW480+TNVs 2h
Caco-2+TNVs 2h
C
RT-PCR DDHD1
2^-ΔCt (DDHD1 levels normalized to the housekeeping)
ns
TNVs+DDHD1 siRNA 200 pmol
HiPerFect DDHD1 siRNA 200 pmol
D
SW480+siRNA loaded TNVs
% of growth vs untreated cells
TNVs+scrambled siRNA 200pmol
TNVs+DDHD1 siRNA 200 pmol
TNVs+scrambled siRNA 400 pmol
TNVs+DDHD1 siRNA 400 pmol
